# Gamification of a Low-Fidelity Paper Doll to Teach Primary Survey to Pediatric Residents

**DOI:** 10.7759/cureus.41996

**Published:** 2023-07-17

**Authors:** Anita A Thomas, Hiromi Yoshida, Ashley E Keilman, Brian Burns, Jessica McDade, Tamar Anderson, Jennifer Reid

**Affiliations:** 1 Pediatric Emergency Medicine, University of Washington, Seattle, USA; 2 Pediatric Emergency Medicine, Seattle Children's Hospital, Seattle, USA; 3 Pediatric Emergency Medicine, Seattle Childrens Hospital, Seattle, USA; 4 Pediatric Critical Care Medicine, University of Washington, Seattle, USA; 5 Pediatric Emergency Medicine, University of Washington Medical Center, Seattle, USA

**Keywords:** low resource, simulation, emergency med, pediatrics, skills and simulation training

## Abstract

When critically ill pediatric patients arrive in the emergency department (ED), a rapid physical evaluation is performed in order to systematically evaluate and address life-threatening conditions. This is commonly referred to as the primary survey. At our institution, pediatric residents are frequently tasked with this role, but they have limited training for or experience with this task. Quality improvement review of real resuscitation recordings at our institution revealed delays in initiation and incomplete primary surveys. We sought to utilize gamification to standardize and optimize reproducible training for the primary survey task for pediatric residents using a low-fidelity paper doll model simulation to improve primary survey performance in actual resuscitations.

## Introduction

When critically ill patients present to the pediatric emergency department (ED), prompt identification of life-threatening injuries per Advanced Trauma Life Support (ATLS) guidelines is critical to their stabilization [1]. The initial physical evaluation, commonly referred to as a primary survey, enables healthcare teams to quickly identify and treat life-threatening conditions affecting a patient's airway, breathing, circulation, disability or mental status, and injuries noted on full skin exposure (commonly referred to as ABCDE to correlate as an acronym to airway, breathing, circulation, disability, exposure) [1,2]. Typically, one member of a resuscitation team performs and verbalizes the primary survey, enabling the entire team to intervene. In our tertiary care, in the academic pediatric hospital emergency department, senior pediatric residents perform the primary survey. However, their training and experience varies, which may contribute to different levels of competency with completing the primary survey. Residents learn how to perform the primary survey in a range of educational forums, distributed across their educational curricula. Pediatric resident training includes Pediatric Advanced Life Support (PALS) certification at the start of their first postgraduate year (PGY1) and third postgraduate year (PGY3) of training. In addition, viewing a primary survey demonstration video performed by an experienced provider is recommended but not required, as is a simulation-based workshop prior to starting their PGY2. Intermittent opportunities to practice the primary survey in a mock code curriculum are distributed throughout their residency, where attendance is recommended but not required. Actual patient experience varies depending on the number of resuscitations they have participated in. Our pediatric ED typically sees an average of 160 resuscitations per year. Quality assurance video review of our pediatric ED resuscitations from March 21, 2016 to June 21, 2019 showed that 72.2% of resuscitations included a complete primary survey, frequently with delays from patient arrival to survey initiation and completion. We sought to improve this number via low-fidelity simulations utilizing gamification given that it has been shown to improve motivation and engagement within graduate medical education [3-5]. Our goal was to create an engaging, time- and resource-efficient primary survey simulation, scaffolded into existing training, which increased the efficiency and quality of primary surveys in patient resuscitations.

## Technical report

Methods

**Figure 1:**
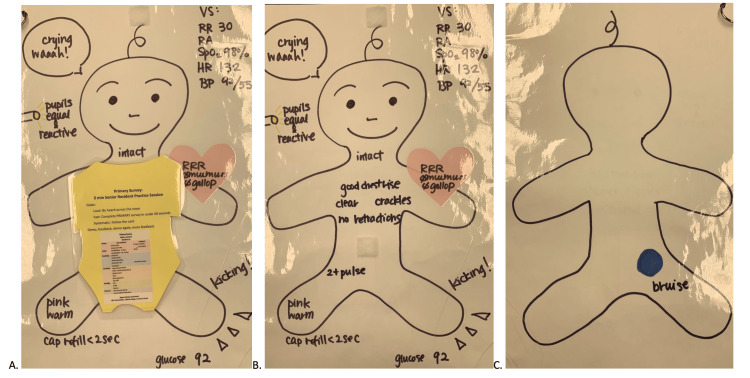
A. Paper doll with instructions; B. front of the paper doll; C. back of the paper doll Figure 1A: Low-fidelity paper doll survey with a “shirt” with instructions and an overview of the primary survey. Figure 1B: Front of the low-fidelity paper doll survey without the “shirt." Figure 1C: Back of the paper doll with a physical exam finding, demonstrating the importance of a full survey. VS: vital signs; RR: respiratory rate; RA: room air, SpO2: oxygen saturation; HR: heart rate; BP = blood pressure; RRR: regular rate and rhythm; Ø: no; Cap: capillary; glucose: 92 mg/dL

A low-fidelity paper doll simulator was developed in conjunction with divisional ED content experts and representatives from graduate medical education (Figure 1). The two-dimensional drawing of a pediatric paper doll patient consisted of a laminated piece of paper, with a drawing of a baby’s ventral and dorsal sides, with physical exam findings on its body. Descriptions of some exam findings were written on the doll (e.g., breath sounds: clear, bilateral), and other exam findings were drawn (e.g., the dorsal side of the doll has a picture of a bruise that could be visualized if the backside of the patient is examined). In addition, the paper doll had removable instructions in the form of the paper doll’s shirt for a facilitator (directions for the simulation session and primary survey job aide) plus a primary survey job aide for a learner that was made available in a small format to be added to identification badge holders (see Appendix). The simulator was placed in the ED resuscitation room in July 2019. 

From July 2019 to June 2020, senior pediatric residents (PGY2-PGY3) received standard training, including watching the recommended video, pre-PGY2 year practice sessions, and various practice opportunities. In addition, they had the opportunity for individual simulation sessions with the paper doll simulator during their pediatric emergency medicine (PEM) rotation for the 2019-2020 academic year. Supervisors included PEM physicians or fellows who were in clinical staffing. Supervising PEM physicians were introduced to the paper doll simulation training plan during a bimonthly staff meeting, in less than 10 minutes. Supervisors were reminded at three subsequent bimonthly staff meetings and via weekly e-newsletter reminders about the simulation training format and expectations. Training sessions, intended to last five minutes or less, could be initiated by either the resident or supervisor. During the session, the resident would call out the primary survey on the paper doll simulator by referring to the information provided on the doll. They would then receive formative feedback from the supervisor regarding their clarity and audibility and repeat the process as needed so that the residents could gain competency in their survey skills.

Senior residents were sent email descriptions of the simulation session and the expectation that they would complete the simulation within the first week of their PEM rotation. To track completion, the residents were asked to email a “selfie” photograph of themselves, the supervising physician, and the paper doll to the pediatric chief residents. Using gamification as an engagement strategy, supervising PEM physicians were incentivized to teach the greatest number of senior pediatric residents per month with a small prize (e.g., coffee gift card). During two physician staff meetings between July 2019 and January 2020, supervisors with the greatest number of teaching sessions received prizes. The residents were incentivized by having their selfies included in weekly resident newsletters and receiving small prizes, such as a gift card award (e.g., individual who sent the most selfies or completed a selfie during a non-ED rotation). The residents were awarded prizes during two resident meetings between July 2019 and January 2020. Video recorded resuscitations in the PEM resuscitation room continued to be reviewed routinely between July 2019 and January 2020 as per our hospital quality assurance work, with the percentage of primary surveys performed recorded as a continued metric. Resuscitations occurring in other ED rooms were not reviewed, as they do not have video recording capability. The Institutional Review Board (IRB) of Seattle Children's Hospital exempted approval.

Results

There were 38 eligible senior residents rotating in the pediatric ED from July 13, 2019 to January 5, 2020 (see Table 1), with 16/38 participants of seven PGY2s and nine PGY3s. Twenty-one training sessions were facilitated over that time period by 10 eligible supervising physicians out of 45 (3/9 PEM fellows and 7/36 attending physicians) participated, with 11 sessions conducted by fellow physicians. The range of participation per participating supervising physician was one to seven training sessions, with a median of 1 performed per participating supervising physician. Upon routine quality assurance video review of ED resuscitations, primary surveys performed by pediatric residents increased to 92.9% from 72.2% in the preceding time frame, with participation greater in July-August of 2019.

**Table 1:** Demographics of the paper doll primary survey training from July 13, 2019 to January 5, 2020 PGY: post-graduate year; ED: emergency department

Demographic	Result
Participating senior pediatric residents (PGY2 or PGY3)/total number of eligible senior pediatric residents (%)	16/38 (42.1%)
Participating supervising physicians/total number of eligible supervising physicians (%)	10/45 (22.2%)
Participating attending physicians/total number of eligible attending physicians (%)	7/36 (19.4%)
Participating fellow physicians/total number of eligible fellow physicians (%)	3/9 (33.3%)
Primary surveys performed by pediatrics residents in ED resuscitations from January 5, 2019 to July 12, 2019 (%)	72.2%
Primary surveys performed by pediatrics residents in ED resuscitations from July 13, 2019 to January 5, 2020 (%)	92.9%

## Discussion

Our low-fidelity paper doll training modality incorporates strategies from adult learning theory to engage participants in an incentivized educational activity. This low-resource intervention, both for supplies and time required of trainees and instructors, is translatable to a range of resource- and time-challenged settings. During the study period, we witnessed improved performance of the primary survey in actual patient care. Resident participants provided positive feedback regarding this educational modality. Over time, this program faced challenges. With the onset of the COVID-19 pandemic immediately after our study period, like with many other residency programs, graduate medical education was disrupted [6]. Standard resuscitation curricula elements, such as PGY-2 pediatrics, practice sessions and the mock code curricula were suspended for a period of time, reducing exposure and experience. Fewer administrative resources were available to remind residents and supervising physicians to conduct training sessions or track completion of training. In-person resident and faculty meetings, where the program was promoted were suspended.The perception of time frame while working clinical shifts and the emotional strain of the pandemic reduced motivation for educational endeavors in general. As the pandemic has progressed, desire for and commitment to resident education has evolved. This simulated low-fidelity paper doll educational tool, with its ability to be incorporated into clinical training time, for succinct periods, has been re-incorporated into resident education and resident orientation, including an introduction to the paper doll survey, which has just gone back live in person as of December 2022. Moving forward, we hope to improve both supervising physician and pediatric resident participation through more frequent and targeted reminders and increasing gamification incentives (e.g., create a scoreboard for PGY-2s vs. PGY3s).

The limitations of this study include that it was conducted in a single center; the paper doll was included as a part of a bundle of educational activities, including an optional video; the impact on patient outcomes was not assessed; and the paper doll was not compared to other educational modalities. This low-resource educational modality is feasible and generalizable to other educational settings, levels of learners, and areas of clinical content, and it warrants further study for future refinement and individual usability to increase use.

## Conclusions

Our low-fidelity paper doll training modality incorporates strategies from adult learning theory to engage pediatric resident participants in a gamified educational activity. It may improve performance in the clinical setting and can be trialed in other hospital settings with other trainees, particularly in low-resource and time-constrained settings and in COVID-influenced educational settings requiring shorter educational sessions that can be incorporated into the existing clinical training time. Future studies are needed to assess impact on clinical outcomes.

## Figures and Tables

**Figure 2:**
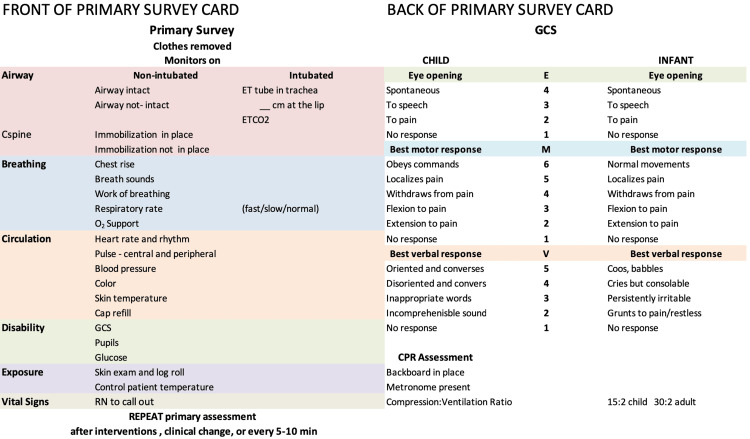
Primary survey job aid (front/back) The primary survey job aid provided to pediatrics residents and sized to fit inside identification badge holders for ease of access. This primary survey representation was adapted from the Pediatric Advanced Life Support and Advanced Trauma Life Support. ET: end tidal; cm: centimeters; ETCO2: end-tidal carbon dioxide; O2: oxygen; cap: capillary; GCS: Glasgow Coma Scale; RN: registered nurse; CPR: cardiopulmonary resuscitation
